# Comparative Analysis of Physiological, Enzymatic, and Transcriptomic Responses Revealed Mechanisms of Salt Tolerance and Recovery in *Tritipyrum*

**DOI:** 10.3389/fpls.2021.800081

**Published:** 2022-01-05

**Authors:** Ze Peng, Yiqin Wang, Guangdong Geng, Rui Yang, Zhifen Yang, Chunmiao Yang, Ruhong Xu, Qingqin Zhang, Kaleem U. Kakar, Zhenhua Li, Suqin Zhang

**Affiliations:** ^1^College of Agriculture, Guizhou University, Guiyang, China; ^2^Research Institute of Pepper, Guizhou Academy of Agricultural Sciences, Guiyang, China; ^3^Guizhou Subcenter of National Wheat Improvement Center, Guiyang, China; ^4^Department of Microbiology, Faculty of Life Sciences and Informatics, Balochistan University of Information Technology, Engineering and Management Sciences, Quetta, Pakistan

**Keywords:** wheat, salinity tolerance, growth, antioxidases, osmoregulators, molecular response

## Abstract

Salt stress results in the severe decline of yield and quality in wheat. In the present study, salt-tolerant *Tritipyrum* (“Y1805”) and salt-sensitive wheat “Chinese Spring” (“CS”) were selected from 121 wheat germplasms to test their physiological, antioxidant enzyme, and transcriptomic responses and mechanisms against salt stress and recovery. 56 chromosomes were identified in “Y1805” that comprised A, B, and D chromosomes from wheat parent and E chromosomes from *Thinopyrum elongatum*, adding to salt-tolerant trait. Salt stress had a greater inhibitory effect on roots than on shoots, and “Y1805” demonstrated stronger salt tolerance than “CS.” Compared with “CS,” the activities of superoxide dismutase and catalase in “Y1805” significantly increased under salt stress. “Y1805” could synthesize more proline and soluble sugars than “CS.” Both the net photosynthetic rate and chlorophyll a/b were affected by salt stress, though the level of damage in “Y1805” was significantly less than in “CS.” Transcriptome analysis showed that the differences in the transcriptional regulatory networks of “Y1805” were not only in response to salt stress but also in recovery. The functions of many salt-responsive differentially expressed genes were correlated closely with the pathways “peroxisome,” “arginine and proline metabolism,” “starch and sucrose metabolism,” “chlorophyll and porphyrin metabolism,” and “photosynthesis.”

## Introduction

Wheat is a global food crop that is fundamental to human civilization. Over 759 million metric tons were consumed in the 2020/2021 marketing year (Ahmad et al., 2016). Soil salinity is one of the most serious types of abiotic stress, known for its detrimental effects on plant growth, development, and productivity, it leads to significant crop yield losses. Soil salinity is one of the most damaging abiotic stresses and a major global problem in modern agriculture (Isayenkov and Maathuis, 2019). Among different factors, salt stress primarily induces higher osmotic pressure in soil solutions due to the accumulation of salt. This results in the impairment of crop growth, overall development, and production. Studies on plant abiotic stress management have shown that both seed germination and subsequent seedling emergence are inhibited by salt stress because of retarded water absorption (Sohrabikertabad et al., 2012; Shu et al., 2017; Li et al., 2020; Zeng et al., 2021). Additionally, a reduction in leaf areas, stomatal conductance, plant heights, and shoot and root weights were observed under salt stress (Mahmood and Quarrie, 1993; Qureshi and Daba, 2019). Globally, about 6% of the soil surface is affected by salt stress, especially in arid and semi-arid regions, where there is insufficient rainfall to filter salt from the upper layers of the soil (Rai et al., 2018). Research has shown that 50% of the world’s arable land will be lost by the middle of the 21st century due to increasing salinization (Goyal et al., 2016). Therefore, the improvement and use of saline soil are considered important for the sustainable development of agriculture. This necessitates a search for plants that are tolerant to salt stress in addition to providing food.

Salt stress induces many physiological responses in plants, mainly through ion stress, osmotic stress, and oxidative stress (Yang and Guo, 2018; Sikder et al., 2020). For instance, some initial responses, such as membrane rupture, accumulation of compatible solutes, and reduction of carbon assimilation, are stimulated by osmotic stress. Similarly, Na^+^ and Cl^−^ are absorbed by plants through transporters and ion channels, resulting in ion toxicity and nutrient imbalance (Han et al., 2015). In addition, the levels of reactive oxygen species (ROS) in plant cells increase, leading to oxidative stress, lipid peroxidation, membrane degradation, and damage to DNA and proteins (Arif et al., 2020). Under salt stress, plants respond through osmotic regulation, ion homeostasis, hormone regulation, photosynthesis, and antioxidant defense systems (Mushtaq et al., 2020; Zhu et al., 2021). At the cellular level, the “salt overly sensitive” (SOS), “mitogen-activated protein kinase,” and “abscisic acid (ABA) signal transduction” pathways are considered to be related to plant salt tolerance (Mishra et al., 2006; Huang et al., 2012; Zhu, 2016). Transporters, such as SOS1, high-affinity K^+^ transporters, and Na^+^/H^+^ and K^+^/H^+^ exchangers, have been found to participate in K^+^ and/or Na^+^ homeostasis and stress tolerance in *Arabidopsis* (Shi et al., 2000; Baxter et al., 2010; Zhu, 2016). The molecular mechanism of plant salt tolerance is very complex, and a large amount of data is required to explore its regulatory network. Recently, genomic, transcriptomic, proteomic, and metabolomic methods have been used to predict regulatory genes and signal pathways in *Arabidopsis* and other species (Abdel-Farid et al., 2020; Han et al., 2020; Yan et al., 2021).

The E genome species (e.g., halophile wheatgrass *Thinopyrum elongatum*) of the *Triticeae* possess salt-tolerant properties, making them invaluable sources for genetic variation and the improvement of wheat crops (Jauhar, 1990; Munns and Tester, 2008; Margiotta et al., 2020). *Tritipyrum* derived from the wide crosses of *Triticum* and *Thinopyrum* displays salt tolerance (Omielan et al., 1991; Yuan and Tomita, 2015). The amphiploid from a cross between common wheat “Chinese Spring” (“CS”) and salt-tolerant *T. elongatum* (Host) Löve, greatly outperformed “CS” in grain yield, biomass, and other characters under salt stress. The presence of the E genome of wheat was associated with the exclusion of Na^+^ and C1^−^ and inclusion of K^+^ as well as retranslocation of K^+^. In the current work, the potential salt tolerance in salt-tolerant *Tritipyrum* (“Y1805”) was studied with an objective of understanding the physiological, biochemical, and molecular mechanisms of salt tolerance in *Tritipyrum* hybrids. The results of this work will provide valuable information for the breeding and improvement of salt-tolerant wheat.

## Materials and Methods

### Plant Materials

A total of 121 germplasms were screened, including cultivars from common wheat, the progeny of wide cross and rye. Two wheat varieties, “Y1805” (salt-tolerant *Tritipyrum*) and “CS” (salt-sensitive common wheat “Chinese Spring”) were used in this investigation (Omielan et al., 1991; Guo et al., 2012; Yuan and Tomita, 2015). “Y1805” is a stable progeny from a wide cross between common wheat and *T. elongatum*.

### Preliminary Screening of Materials

For the current work, complete and uniform wheat seeds were thoroughly selected, disinfected with 70% alcohol for 1 min, and then washed repeatedly with sterilized distilled water. The seeds were soaked in Petri dishes with two layers of wet filter paper. After germination, seedlings with the same growth vigor were selected and planted in a 24-hole tray with peat substrate. Five seedlings were planted in each hole (36 cm^2^ per hole), and three holes were sown for each germplasm. On the 7th day after germination, seedlings were subjected to salt stress treatment for 14 days by watering NaCl solution (180 mM), and sterilized distilled water was used as control. Plant growth was maintained at 24/18°C for a 16/8 h light/dark photoperiod with an irradiance of 400 μmol m^−2^ s^−1^. The experiment was repeated three times. The roots were cleaned to remove peat substrate and then extracted from the root–stem interface. Root fresh weight (RFW), shoot fresh weight (SFW), root dry weight (RDW), and shoot dry weight (SDW) were measured for all 121 germplasms. RDW and SDW measurements were taken after that the roots and shoots were oven-dried at 90°C for 48 h. We evaluated RFW, SFW, RDW, and SDW of 121 germplasms through membership function (Ding et al., 2018), obtained the membership function value of each germplasm, and conducted cluster analysis based on within-group linkage and squared Euclidean distance (Hunter and Mccoy, 2004).

### Sequential GISH-FISH Analysis

Genomic *in situ* hybridization (GISH) and fluorescence *in situ* hybridization (FISH) were performed as described by Kato et al. (2004) and Han et al. (2006). The labeled genomic DNA of *T. elongatum* was used as a probe for GISH analysis. Oligo-pAs1-1, Oligo-pAs1-3, Oligo-pAs1-4, Oligo-pAs1-6, Oligo-AFA-3, Oligo-AFA-4 (red), Oligo-pSc119.2-1, and (GAA)_10_ (green) probes were used according to Du et al. (2017). Oligonucleotide probes were synthesized by Shanghai Invitrogen Biotechnology Co. Ltd. (Shanghai, China). The slides were mounted in Vectashield antifade solution containing 4′-6-diamino-2-phenylindole (DAPI; Vector Laboratories Inc., Burlingame, CA, United States). A fluorescence microscope (BX60, Olympus Corp., Tokyo, Japan) fitted with a Spot CCD camera was used to capture hybridization signals. The images were compiled with CellSens Vers.1.5 Imaging software (Olympus Corp.).

### Growth Conditions and Stress Treatment

The seeds of “Y1805” and “CS” were germinated in a growth chamber at 24/18°C and relative humidity of 75%. The seedlings were transferred to 1/2 Hoagland solution and incubated under 16/8 h of light/dark with an irradiance of 400 μmol m^−2^ s^−1^ and germinated at the same temperature and humidity as in the chamber. The culture solution was refreshed every 3 days. On the 14th day (two-leaf stage), uniformed-sized roots and tops were collected as the first samples (T1 stage, no salt stress). Then, 250 mM NaCl salt stress treatment (NaCl +1/2 Hoagland solution) was applied. The second and third samples were selected at 5 h (T2 stage) and 24 h (T3 stage) respectively after salt stress treatment. Afterward, salt stress was removed, and the materials were recovered. The fourth and fifth samplings were performed at 1 h (R1 stage) and 24 h (R2 stage) after recovery. These sampling stages were chosen according to typical phenotypic observations. Normal (1/2 Hoagland solution) cultured materials were used as parallel controls. All the samples were immediately frozen in liquid nitrogen after sampling and stored at −80°C for physiological and biochemical analysis, transcriptomic analysis, and confirmational qRT-PCR.

### Analysis of Growth, Physio-Biochemical Parameters

#### Analysis of Growth

Root dry weight and shoot dry weight were determined after roots and seedlings were oven-dried at 90°C for 48 h. Seminal root length (RL) and shoot height (SH) of “Y1805” and “CS” samples were measured at each stage.

#### Analysis of Photosynthesis

The chlorophyll contents of leaves were measured according to an assay by Arnon (Jain and Gadre, 1997). Chlorophyll a (Chl-a) and chlorophyll b (Chl-b) were extracted by 80% acetone, and their contents were read at 645 and 663 nm, respectively. The net photosynthetic rate (Pn) of leaves was measured using an LI-6400 XT portable photosynthesis system (Li-Cor Inc., Lincoln, NE, United States).

#### Analysis of Enzyme Activities

##### Enzyme Extraction

The subsequent biochemical and physiological parameters were measured using the roots of the wheat plants. Enzymes were extracted using the method of Xu et al. (2021). Briefly, 0.50 g of roots were collected and ground in 6 mL of 50 mM phosphate buffer (pH 5.5) containing 5 mM mercaptoethanol, 1 mM EDTA, and 1% polyvinylpyrrolidone in an ice bath, and then centrifuged at 3,550 *g* and 4°C for 10 min. The supernatant was used as the extract.

##### Spectrophotometric Assay

*SOD (EC1.15.1.1)*. Total SOD activity was assayed by monitoring the inhibition of nitro blue tetrazolium (NBT) photochemical reduction by using the method by Jiang and Zhang (2001). The 3 mL reaction mixture contained 50 mM potassium phosphate buffer (pH 7.8), 13 mM methionine, 75 μm NBT, 2 μm riboflavin, 0.1 mM EDTA, and 100 μL enzyme extract. The reaction mixtures were illuminated for 15 min at a light intensity of 400 μmol m^−2^ s^−1^. One unit of SOD activity was defined as the amount of enzyme required to cause 50% inhibition to the NBT reduction as monitored at 560 nm.

*CAT (EC 1.11.1.6)*. The CAT activity was measured by its ability to decompose H_2_O_2_ (Aebi, 1984). The 3 mL mixture consisted of 50 mM potassium phosphate buffer (pH 7.0), 10 mM H_2_O_2_, and 200 μL of enzyme extract. The absorbance was observed at 240 nm.

##### Analysis of Osmoregulators

*The Proline Content*. A modified method by Bates et al. was used to measure the proline content (Bates et al., 1973). Briefly, 0.5 g of roots were extracted with 10 mL of 3% sulfosalicylic acid. After centrifugation of homogenate at 10,000 *g* for 10 min, 0.5 mL of the supernatant was added into 0.5 mL of ninhydrin and 0.5 mL of glycol acetic acid. The reaction was carried out at 100°C for 30 min. After cooling in an ice bath, 1 mL toluene was added and the absorbance of the mixture was measured at 520 nm. The proline content was determined according to a standard curve.

*Soluble Sugar Content*. The soluble sugar content was determined by the anthrone sulfuric acid method (Leyva et al., 2008). About 0.1 g anthrone was dissolved in 100 mL of 98% sulfuric acid and stored away from light. Then, 150 μL anthrone reagent was added into 50 μL of sample extraction solution. The samples were incubated at 100°C for 20 min. After cooling to room temperature, the absorbance was measured at 620 nm.

### Transcriptome Analysis

The data obtained from phenotypic, physiological, and biochemical analysis were used to choose the two key sampling stages (T2 and R1) of “Y1805” and “CS” for further transcriptome analysis. Total RNA from the roots was extracted using TRIzol reagent (Thermo Fisher Scientific, Waltham, MA, United States) following the manufacturer’s instructions, and then treated with TaKaRa RNase-free DNase I for 30 min. NanoDrop 1000 spectrophotometer was used to test the quantity and quality of the extracted RNA. A total of 20 μg RNA was used for cDNA library construction and transcriptome sequencing (BGISEQ-500) at the Beijing Genome Institute. After data filtering, clean reads were obtained and then compared with the reference genome (wheat “CS,” AABBDD) by using HISAT2 (version 2.1.0) software. Finally, fragments per kilobase of exon model per million mapped fragment (FPKM) values were calculated by RSEM (version 1.2.8), and the level of gene expression was quantified (Bates et al., 1973).

### DEG Identification and Bioinformatics Analysis

The FPKM method was used to detect differentially expressed genes (DEGs) among the treatment and control samples (Trapnell et al., 2010). To compute the significance of the differences in gene expression levels, a false discovery rate (FDR) method was used to determine the threshold of the value of *p*. Then, the genes potentially regulated by treatment were identified using an FDR threshold <0.01, value of *p* < 0.001, and absolute log_2_fold change value (|log_2_FC|) > 1 between all three salt-treated and three salt-free samples using DESeq software (Anders and Huber, 2010). The Phyper function in the R package was used for enrichment analyses of Gene Ontology (GO) and Kyoto Encyclopedia of Genes and Genomes (KEGG).

### Quantitative Real-Time PCR Validation

The candidate DEGs were verified by qRT-PCR. Total RNA extracted from root tissue was reverse transcribed into cDNA using PrimeScript^™^ RT reagent Kit with gDNA Eraser (Perfect Real-Time, Takara Bio, Dalian, China). An ABI step-one fluorescent quantitative PCR system (Applied Biosystems, Foster, CA, United States) was used to analyze the DEGs. The relative expression levels were calculated with the 2^−ΔΔCt^ method in three biological replications and three technical replications (Depuydt and Hardtke, 2011), and actin-7 (Accession No. LOC123057740) was used as the internal control.

### Data Analysis

A randomized complete block design with three replications was employed in the experiments. The results were expressed as the mean and standard deviation of three separate replicates. Statistical software (SPSS 20.0, IBM Corp., Armonk, NY, United States) and graphics software (Origin 2018, OriginLab, Northampton, MA, United States) were used for the data analysis and subsequent representation. ANOVA and Duncan’s multiple range tests were used to compare means and determine the differences between means at a significance level of *p* < 0.05. Pearson’s correlation analysis of binary variables was performed, and two variables were considered significantly correlated at the *p* < 0.05 level. The cluster analysis was carried out by using the square Euclidean distance on the average linkage between groups.

## Results

### Preliminary Salt-Tolerant Screening of Materials

**Figure 1:**
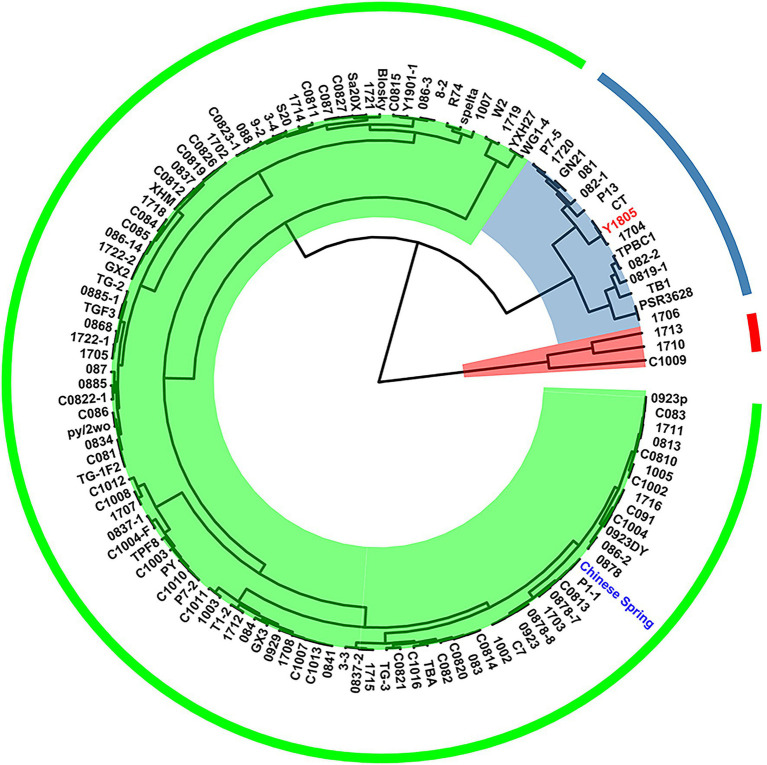
Clustering analysis based on salt-tolerant evaluation in wheat germplasms.

We determined RFW, SFW, RDW, and SDW of 121 germplasm resources as indicators for salt tolerance. As shown in Figure 1, these germplasms were divided into three categories based on their salt tolerance indices (value for the sodium chloride treated plant/value for the control). Of these, “Y1805” was classified as salt-tolerant germplasm. “Y1805” is a distant hybrid of wheat, widely used in wheat breeding, and has been proven to be salt-tolerant. Whereas “CS” was classified as salt-sensitive germplasm (Figure 1), “CS” is considered an important variety in wheat genetics, and a proven salt-sensitive cultivar.

### Sequential GISH-FISH Analysis of *Tritipyrum* “Y1805”

**Figure 2:**
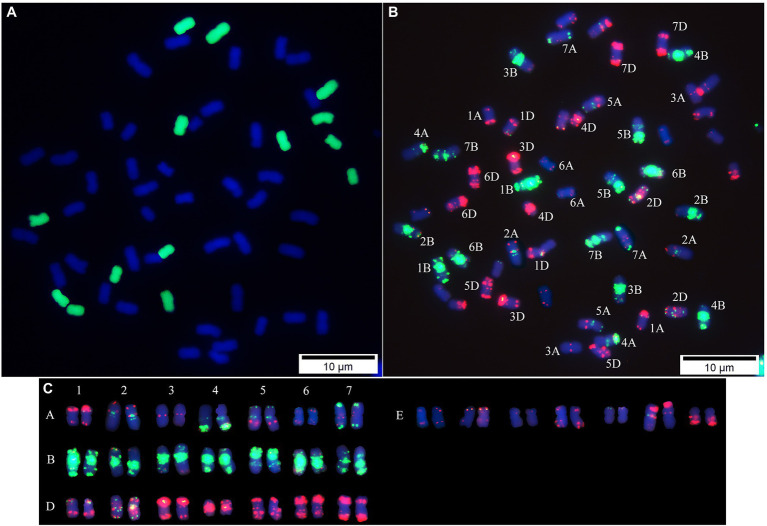
Sequential GISH-FISH analysis of *Tritipyrum* (“Y1805”). **(A)** GISH patterns of chromosomes of “Y1805” using labeled genomic DNA of *Thinopyrum elongatum* as the probe (green). **(B)** FISH patterns of “Y1805” chromosomes using Oligo-pAs1-1, Oligo-pAs1-3, Oligo-pAs1-4, Oligo-pAs1-6, Oligo-AFA-3, Oligo-AFA-4 (red), Oligo-pSc119.2–1 and (GAA)_10_ (green) probes, counterstained with DAPI (blue). **(C)** FISH karyotypes of “Y1805”.

Using the labeled genomic DNA of *T. elongatum* as a probe, GISH analysis detected a group of chromosomes with a green signal in “Y1805” (Figure 2A), which confirmed that the alien chromosomes belonged to *T. elongatum*. “Y1805” chromosomes could be effectively distinguished by FISH analysis. It was demonstrated that “Y1805” not only had A, B, and D chromosomes from wheat parent but also contained a set of alien chromosomes that originated from the E genome of *T. elongatum* (Figure 2). For the E genome chromosomes, the red fluorescence signals were greater than the green signals and had a different karyotype pattern compared with the A, B, and D chromosomes of common wheat. Thus, both GISH and FISH analysis demonstrated that “Y1805” not only had three sets of chromosomes from common wheat but also had an E genome of chromosomes from *T. elongatum*. “Y1805” and “CS” were selected for subsequent molecular biology experiments.

### Growth Responses to Salt Stress and Recovery in Two Wheat Varieties

**Figure 3:**
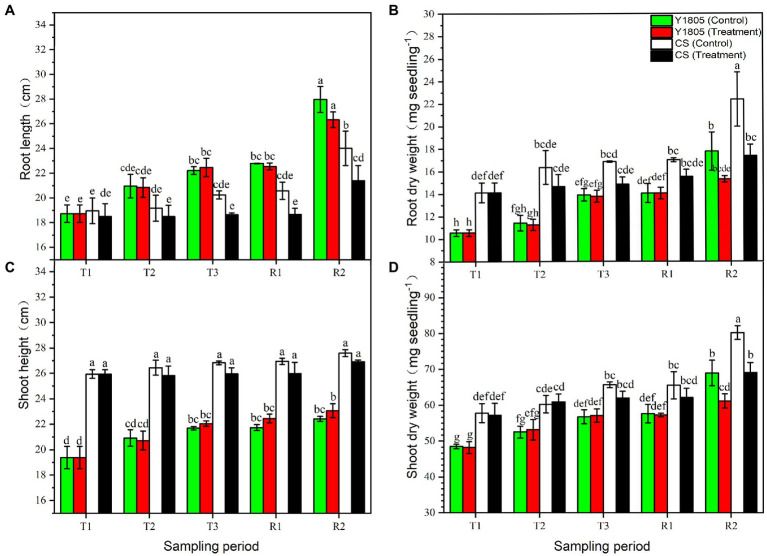
Effect of salt stress and recovery on growth parameters of two wheat varieties. **(A)** Root length. **(B)** Root dry weight. **(C)** Shoot height. **(D)** Shoot dry weight. Bars indicate means with SDs (*n* = 3). Values with different letters are significantly different at *p* < 0.05.

The growth of crops under salt stress depends on their salt tolerance and resilience after the recovery (Guo et al., 2015). Our analysis of the growth indices indicated an inhibitory effect of salt stress in “CS” at the R2 stage, which was significantly less in “Y1805,” especially in roots (Figure 3). Salt stress had a greater inhibitory effect on the root than the leaves (Figure 3). RL of salt-tolerant “Y1805” at the T3, R1, and R2 stages was significantly greater than the “CS” under both salt stress and recovery conditions, and there was no significant difference between the treatments and controls at each stage (Figure 3A). Compared with the control, the RL of salt-sensitive “CS” was reduced at each stage. A reduction in RL of 10.84% was observed at the R2 stage after recovery (Figure 3A). At the R2 stage, the RDW of recovered “Y1805” tissues did not show a significant difference compared with the control (Figure 3B) whereas that of the “CS” decreased significantly by 22.35% (Figure 3B). Relative to controls, no significant differences were observed in SH of “Y1805” at each stage of the salt stress and recovery, except for a slight increase at the R2 stage (Figure 3C). On the other hand, the SH of “CS” was comparatively higher, yet exhibited no significant difference compared with the controls at each stage (Figure 3C). Similar results were noted for SDW. At the R2 stage, the SDW of “Y1805” and “CS” decreased by 11.30 and 13.83%, respectively (Figure 3D). At the R2 stage, the absolute values of RDW and SDW in “Y1805” were less than those of the “CS” under normal and treatment conditions because of their different genotypes. Overall, RL, RDW, and SDW of “CS” were significantly lower than those of the controls at the R2 stage, whereas there was no significant difference in RL and RDW shown by “Y1805,” and an even slightly higher SH than that of the control, which may be attributed to differences in their mechanisms toward salt stress and recovery.

### Physio-Biochemical Responses to Salt Stress and Recovery in Two Wheat Varieties

#### Analysis of Antioxidant Enzyme Activities

**Figure 4:**
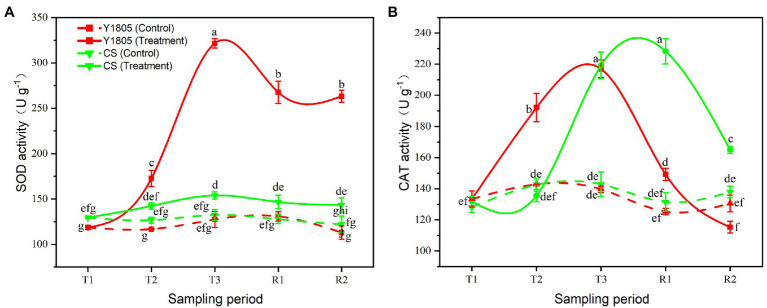
Effect of salt stress on antioxidant enzyme activities in two wheat varieties. **(A)** SOD activity. **(B)** CAT activity. Bars indicate means with SDs (*n* = 3). Values with different letters are significantly different at *p* < 0.05.

Antioxidant enzyme activity is an important index to measure the ability of plants to resist salt stress. Superoxide dismutase (SOD) and catalase (CAT) enzymes help plants in the defense against possible oxidative damage (Agami, 2014). Here, we assessed the activities of SOD and CAT in two wheat varieties subjected to salt stress and recovery. Compared with the controls, the activities of SOD and CAT sharply increased in “Y1805” during salt stress and recovery stages, whereas only CAT activity was dramatically increased in “CS” (Figure 4). The SOD activity of “Y1805” began to rise sharply at the T2 stage and peaked at the T3 stage under salt stress, which was ~2.16 and ~2.60-fold higher than the “CS” and control, respectively (Figure 4A). The SOD activity in “Y1805” was maintained at a higher level during the R1 and R2 recovery stages after a gradual decline. The SOD activity of “CS” increased slowly during salt stress, and the difference between the treatment and control was significant only at the T3 stage. The CAT activity of “Y1805” increased significantly from the T2 stage and reached a peak at the T3 stage, and then decreased rapidly after recovery (Figure 4B). This trend demonstrated that “Y1805” enhanced CAT activity rapidly under salt stress to protect the cells from damage caused by ROS, and reduced quickly after recovery. In “CS,” the CAT activity increased slowly during salt stress, peaked at the R1 stage, and dropped afterward. The general trend of the two species was similar, the activities of antioxidant enzymes began to rise after salt stress, and began to decline during the recovery process, which indicated that wheat could cope with salt stress by adjusting the activities of antioxidant enzymes.

#### Analysis of Osmoregulation

**Figure 5:**
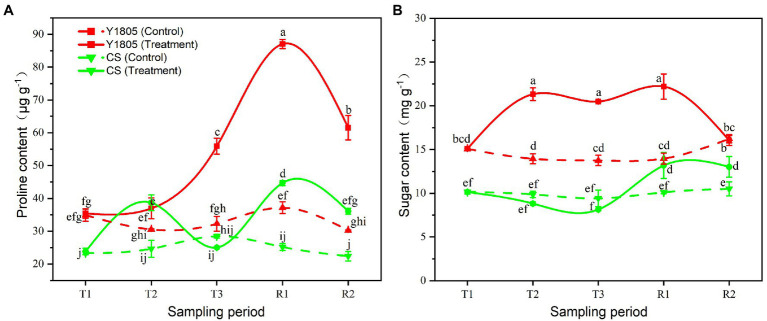
Effect of salt stress and recovery on osmoregulator levels in two wheat varieties. **(A)** Proline content. **(B)** Sugar content. Bars indicate means with SDs (*n* = 3). Values with different letters are significantly different at *p* < 0.05.

Proline and soluble sugar are important regulators for the ability of plants to cope with osmotic stress caused by salt stress. Our results indicated that the salt stress and recovery responses of “Y1805” were stronger and faster than those of “CS.” During development under normal conditions, and under stress and recovery, levels of osmoregulants were generally higher in “Y1805” than in “CS” (Figure 5). Under salt stress, the contents of both proline and soluble sugar in “Y1805” increased significantly from the T2 stage, peaked at the R1 stage, and then gradually declined. The proline content of “Y1805” began to rise at the T2 stage under salt stress and reached maximum at the R1 stage, which was ~2.00 and ~2.46-fold higher than “CS” and the control, respectively (Figure 5A). Similarly, the soluble sugar content in “Y1805” increased rapidly from the T2 stage, kept stable until the R1 stage, and returned to the control level at the R2 stage (Figure 5B). The soluble sugar content of the “Y1805” was ≥1.40-fold higher than the “CS” and control at the T2, T3, and R1 stages. In “CS,” the proline content increased from the T1 to T2 stages, suddenly declined to a slightly lower level than the control at the T3 stage, and increased at the R1 stage. In addition, the soluble sugar level of “CS” was generally lower than the control during the T2 and T3 stages but gradually increased at the R1 stage.

### Analysis of Chlorophyll Content and Photosynthesis

**Figure 6:**
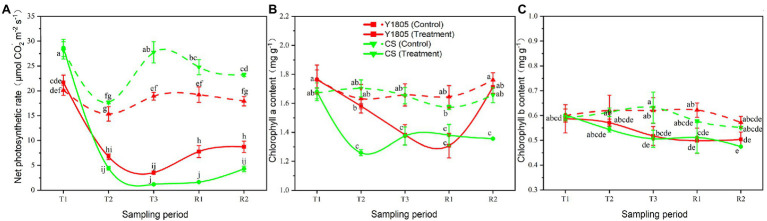
Effect of salt stress and recovery on chlorophyll contents and photosynthetic rate in two wheat varieties. **(A)** Net photosynthetic rate. **(B)** Chlorophyll a content. **(C)** Chlorophyll b content. Bars indicate means with SDs (*n* = 3). Values with different letters are significantly different at *p* < 0.05.

Salt stress is harmful to the photosynthesis of plants (Mbarki et al., 2018). In this experiment, we studied the impact of salt stress and recovery on the photosynthetic activities of two wheat varieties by measuring the levels of Chl-a, Chl-b, and Pn (Figure 6). The results showed that salt stress greatly affected the photosynthesis processes of “Y1805” and “CS,” indicated by lower levels of Chl-a and Chl-b, and Pn than the control samples. However, the Chl-a content of “Y1805” was significantly higher than the “CS” after salt stress at the T2 stage and recovered well at the R2 stage. The Pn of both wheat varieties started to decline at the T2 stage, reached a minimum level at the T3 stage, and started to recover during the R1 and R2 stages (Figure 6A). At the T2 stage, the respective Pn of “Y1805” and “CS” decreased significantly by 56.13 and 75.07% under salt stress compared with the controls. The Pn of “Y1805” was significantly higher than that of “CS” at the R1 and R2 stages showing that “Y1805” could maintain a higher Pn under salt stress, and recover more rapidly than “CS.” Similarly, the Chl-a content of both “Y1805” and “CS” fell during salt stress (T2 and T3). The Chl-a content of “Y1805” at the T2 stage was higher than that of “CS” and recovered well at the R2 stage (Figure 6B). At the T2 stage, the Chl-a content of “CS” decreased significantly by 26.07% compared with “Y1805” under salt stress. After recovery, Chl-a content in “Y1805” rapidly returned to the control level at the R2 stage; however, little change occurred in “CS.” These observations suggest that “Y1805” had a stronger Chl-a resilience ability after recovery, but “CS” suffered irreversible damage due to salt stress. Lastly, “Y1805” and “CS” showed a similar pattern of salt stress/recovery and Chl-b contents, that is, the Chl-b levels of both varieties dropped during salt stress and changed little after recovery (Figure 6C). The lower values of chlorophyll and Pn in “Y1805” and “CS” compare with those of the control under salt stress and the recovery period, indicating that salt stress inhibited the photosynthesis of the two wheat varieties, but “Y1805” was significantly less affected than “CS.”

### DEG Identification in Two Wheat Varieties Under Salt Stress and Recovery

**Figure 7:**
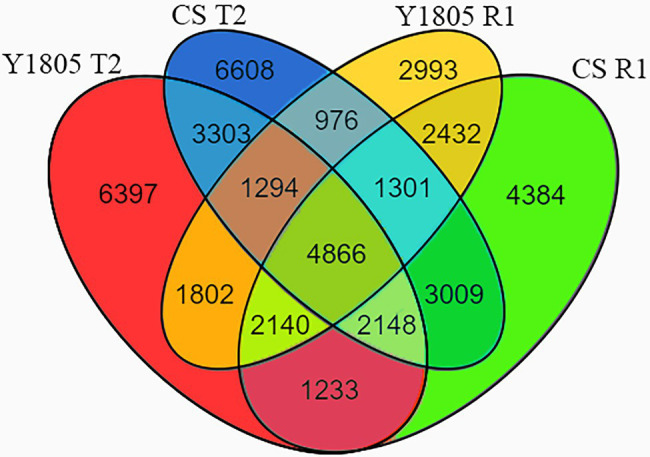
Venn diagrams of DEGs under salt stress and recovery conditions. “Y1805” and “CS” are salt-tolerant and salt-sensitive wheat varieties, respectively. T2 and R1 indicate salt stress and recovery, respectively. False discovery rate (FDR) ≤ 0.01 and absolute log_2_fold change value (|log_2_FC|) > 1 are used as the thresholds to judge the significance of gene expression differences.

To identify salt stress-responsive DEGs, we performed transcriptomic sequencing of root samples of salt-tolerant (“Y1805”) and salt-sensitive (“CS”) germplasms exposed to 250 mM NaCl solution (T2 stage) and after recovery (R1 stage). A total of 112,454 unigenes were obtained from RNA-seq data and 44,886 DEGs were found (Figure 7). Of these DEGs, 30,885 were identified in “Y1805” and 33,694 in “CS,” respectively. Venn diagram analysis indicated that 23,183 and 23,505 unigenes were differentially expressed in response to salt stress in “Y1805” and “CS,” respectively (Figure 7; DEGs at the T2 phase), and 17,804 and 21,513 unigenes were expressed in response to recovery (DEGs at the R1 stage). In “Y1805,” there were 6,397 and 2,993 genes unique to the salt stress and recovery response, respectively, whereas 1,802 “Y1805”-specific DEGs were co-regulated by both salt stress and recovery. Likewise, there were 6,608 and 4,384 “CS”-specific DEGs, regulated under salt treatment and recovery only, while, 3,009 DEGs were co-expressed under both conditions.

### Annotation and GO Enrichment Analysis of Salt/Recovery Responsive DEGs in Two Wheat Varieties

We conducted GO enrichment analysis to characterize the biological functions of the DEGs in “Y1805” and “CS” under salt stress and recovery. The results indicated that the most significantly enriched GO term was markedly greater in “Y1805” (87) than “CS” (69). The top functional terms significantly (*p* < 0.01) enriched in both “Y1805” and “CS” under salt stress or recovery included oxidoreductase, catalytic activity, and amino acid metabolism (Supplementary Tables S1 and S2). The GO terms exclusively enriched in “Y1805” were related to cell cycle, proliferation and differentiation, ion homeostasis, carbohydrate metabolism, nucleoside phosphate binding, protein binding, photosynthesis, ion transport, and protein refolding. The GO terms enriched in “CS” were phenylpropanoid metabolism, organic acid metabolism, amine metabolism, and cellular hormone metabolism.

Under salt stress (T2 stage), 39 GO terms belonging to functional categories of ion homeostasis (9), catalytic activity (7), cellular process (6), photosynthesis (5), homeostatic process (4), signaling (3), transporter activity (2), protein refolding, ion transport, and lipid metabolic activity were exclusively enriched in “Y1805” at value of *Q* < 0.01 (Supplementary Table S3). Among the salt-responsive DEGs in “Y1805,” 138 were associated with “transferase/phosphatase/dehydratase activity,” 86 with “cell wall organization” or “biogenesis/chaperone-mediated protein folding/microtubule bundle formation,” 85 with “ion transport,” followed by “cellular chemical homeostasis” (76), “inorganic/metal ion homeostasis” (40), “photosynthesis-specific metabolic processes” (19), “transporter activity” (11), “protein refolding” (11), “tetraterpenoid metabolic process” (10), and “cell–cell signaling” (3). In “Y1805”-specific DEGs, GO terms for “acid phosphatase activity,” “dihydroxy-acid dehydratase activity,” “S-malonyltransferase activity,” “plant-type secondary cell wall biogenesis,” “chaperone-mediated protein folding,” “microtubule bundle formation,” “chlorophyll metabolic process,” “carotenoid metabolic process,” “inorganic phosphate transmembrane transporter activity,” and “L-ascorbic acid transmembrane transporter activity” were enriched under salt stress. During the recovery process (R1 stage), the “organic substance metabolism” and RNA (LSU-rRNA/ncRNA/tRNA) processing related GO terms were significantly enriched in “Y1805,” which indicated that “Y1805” had abundant gene expression pathways in response to salt stress compared to recovery response (Supplementary Table S4). The common DEGs of salt stress and recovery in “Y1805” were enriched in amino acid metabolism, oxidoreductase, catalytic activity, organic substance metabolism, and cellular process (Supplementary Table S5). There were fewer specific GO terms in “CS” than in “Y1805,” especially in response to salt stress. Under salt stress, 21 GO terms mainly involved in diverse functions of secondary metabolism (61 DEGs), oxidoreductase (83), ion binding (83), amino acid metabolism (26), amine metabolic process (25), structural molecule activity (14), organic substance metabolism (11), transcription (8), and catalytic activity (6) were observed in “CS” (Supplementary Table S6). During the recovery process, GO terms mainly involved in binding (single-stranded RNA binding/organic cyclic compound binding/DNA binding), cellular process, catalytic activity, and organic substance metabolism, were significantly enriched in recovery-specific DEGs of “CS” (Supplementary Table S7). The GO terms associated with oxidoreductase, phospholipid metabolism, amino acid metabolism, organic acid metabolism, and response to stimulus were enriched in “CS” responsive to both salt stress and recovery (Supplementary Table S8).

### Analysis of KEGG Pathways in Salt/Recovery Responsive DEGs in Two Wheat Varieties

Kyoto Encyclopedia of Genes and Genomes enrichment analysis revealed similar pathways in the DEGs of “Y1805” and “CS” under salt stress and recovery, but the number of genes in respective pathways differed (Supplementary Tables S9 and S10). Common pathways, namely, “transcription,” “lipid metabolism,” “folding, sorting, and degradation,” “transport and catabolism,” “signal transduction,” “metabolism of terpenoids and polyketides,” “glycan biosynthesis and metabolism,” “nucleotide metabolism,” “DNA replication and repair,” “metabolism of cofactors and vitamins,” “translation,” “amino acid metabolism,” and “biosynthesis of other secondary metabolites” appeared in both “Y1805” and “CS.” The most significantly (value of *Q* < 0.05) enriched KEGG pathways in “Y1805”-specific DEGs were associated with “folding, sorting, and degradation,” “transport and catabolism,” “carbohydrate metabolism,” and “glycan biosynthesis and metabolism.” There were multiple pathways in “replication and repair,” and their DEGs were more abundant in “Y1805” than “CS.” Compared with “Y1805,” the DEGs of “CS” were more abundant in pathways related to “metabolism of terpenoids and polyketides,” “energy metabolism,” “amino acid metabolism,” and “biosynthesis of other secondary metabolites” (Supplementary Table S10).

Furthermore, KEGG enrichment analysis of “Y1805”-DEGs identified under salt stress found that DEGs were significantly enriched in “metabolism” (carbohydrates, lipids, amino acids, nucleotides, terpenoids and polyketides, and cofactors and vitamins), “transcription,” “translation,” “replication and repair,” “signal transduction,” “environmental adaptation,” “biosynthesis of other secondary metabolites,” “folding, sorting, and degradation,” and “membrane transport” pathways (Supplementary Table S11). We observed that pathways for “biosynthesis of secondary metabolites” and “synthesis and degradation of ketone bodies” were enriched exclusively in “Y1805” that were responsive to both salt stress and recovery (Supplementary Table S12). In addition to the activation of different “cellular processes” and “metabolism” pathways, the salt stress seemed to suppress autophagy-related genes in “CS” (Supplementary Table S13). During the recovery process, “lipoic acid metabolism” pathway was enriched solely in “CS” (Supplementary Table S14).

### Participation of Key Genes in Salt Stress-Related Pathways

We looked for key genes involved in antioxidant enzymes, osmoregulation, and photosynthesis related metabolic pathways under salt stress in “Y1805.” The “peroxisome” (ko04146), “arginine and proline metabolism” (ko00330), “starch and sucrose metabolism” (ko00500), “photosynthesis” (ko00195), and “chlorophyll and porphyrin metabolism” (ko00860) pathways were enriched under salt stress. There were three upregulated genes and four downregulated genes encoding CAT, four upregulated and five downregulated genes encoding SOD in the “peroxisome” pathway (Supplementary Table S15). In the “arginine and proline” pathway, three upregulated genes encoding 1-pyrroline-5-carboxylate synthetase (*BGI_novel_G009739*, *BGI_novel_G019140*, and *TraesCS3D02G357200*), three downregulated genes encoding proline dehydrogenase (*TraesCS1A02G209100*, *TraesCS1B02G223300*, and *TraesCS1D02G212400*), and four upregulated genes encoding prolyl 4-hydroxylase (including *TraesCS2B02G292200*, *TraesCS4B02G228600*, and *TraesCS4D02G229700*) were found. In the “starch and sucrose” pathway, there were four upregulated genes (including *TraesCS4D02G169800*, *TraesCS2D02G175600*, and *TraesCS4A02G140000*) encoding sucrase synthase, six upregulated genes encoding sucrose-phosphate synthase (including *TraesCS3A02G425500*, *TraesCS4A02G225100*, and *TraesCS4B02G091100*), five downregulated *MGAM* (maltase-glucoamylase) genes (including *TraesCS7D02G451800*, *TraesCS7A02G134500*, and *TraesCS7B02G364700*) encoding maltase-glucoamylase, 15 upregulated genes (including *TraesCS2A02G588300*, *TraesCS2B02G595000*, and *TraesCS2D02G570000*) encoding *SacA* of beta fructofuranosidase, 31 *amyA* genes (including *TraesCS2A02G309400*, *TraesCS2B02G183400*, and *TraesCS6B02G349500*) encoding alpha-amylase were downregulated. In the “photosynthesis” pathway, there was one upregulated *PsbC* gene (*BGI_novel_G013282*) encoding photosystem II CP43 chlorophyll apoprotein, two upregulated *PetA* genes (*TraesCS1D02G181700* and *TraesCS5D02G200200*) related to apocytochrome f, and one upregulated *psb27* gene (*TraesCS4D02G172800*) encoding the photosystem II psb27 protein. In the “chlorophyll and porphyrin” pathway, there were two downregulated *chlG*/*bchG* genes (*TraesCS1B02G237700* and *TraesCS1D02G226100*) encoding Chl-a synthase, two upregulated genes (*TraesCS3D02G103900* and *TraesCS5D02G364100*) encoding chlorophyllase, two upregulated *NOL/NYC*1 genes (*TraesCS3A02G151900* and *TraesCS3D02G159800*) encoding chlorophyll (ide) b reductase, three downregulated *CAO* genes (*TraesCS3A02G506200*, *TraesCS3B02G574300*, and *TraesCS3D02G514100*) encoding chlorophyllide a oxygenase, and nine upregulated *SGR/SGRL* (stay green rice/stay green rice like) genes (including *TraesCS6B02G143000*, *TraesCS3A02G447000*, and *TraesCS5B02G320200*) encoding magnesium dechelase.

### Verification of DEGs by qRT-PCR

**Figure 8:**
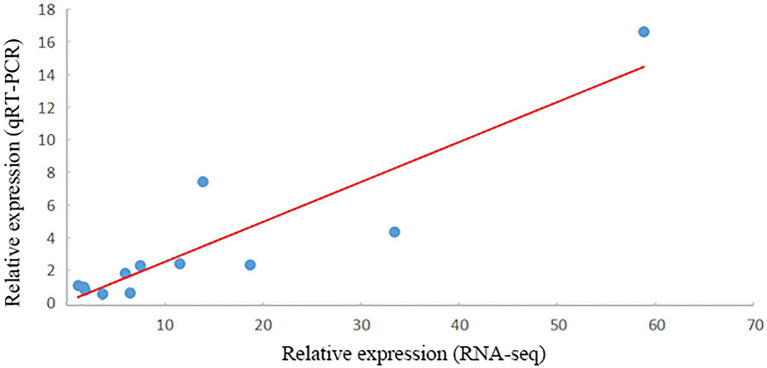
Verification of RNA-seq results by qRT-PCR. The x- and y-axes show the relative expression levels analyzed independently by RNA-seq and qRT-PCR, respectively.

In total, 12 DEGs closely related to salt stress were selected for qRT-PCR analysis. All the gene amplification levels, as assessed by qRT-PCR, agreed well with the RNA-seq patterns, and the correlation between RNA-seq and qRT-PCR showed a positive correlation coefficient (*r* = 0.902, *p* = 6.16E^−5^; Figure 8).

## Discussion

Crop models, such as *Tritipyrum*, are essential tools for assessing the threat of salt stress to local and global food production crops including wheat. In this work, we studied salt-tolerant mechanisms in *Tritipyrum* by assessing the growth, physiological, and antioxidant enzymatic responses of salt-tolerant *Tritipyrum* (“Y1805”) and a salt-sensitive common wheat variety (“CS”). Sequential GISH-FISH technique were used to determine the genome structure and genetic diversity of salt-tolerant hybrid germplasm. Moreover, RNA-seq analysis, GO annotation, KEGG pathway enrichment, and qRT-PCR analyses were used to identify key candidate genes and investigate the molecular mechanisms of salt tolerance and recovery in “Y1805.”

In recent years, RNA-seq analysis based DEGs and candidate gene identification has been used to study salt tolerance in numerous plants, including *Arabidopsis* (Sako et al., 2021), rice (Chandran et al., 2019), bread wheat (Amirbakhtiar et al., 2021), Chinese cabbage (Li et al., 2021), drought/salt-tolerant *Caragana korshinskii* (Li et al., 2016), a model halophytic *Chenopodium quinoa* (Ruiz et al., 2019), *Cenostigma pyramidale* (Frosi et al., 2021), and *Ricinus communis* (Lei et al., 2021). These studies have identified abundant DEGs, candidate upregulated genes, and the functional enrichment of GO terms and pathways related to salt stress responses in plants. Additionally, many physiological and biochemical genes have been verified experimentally to play key roles in the salt tolerance mechanism of plants. Singh et al. found that when the S*bpAPX* gene encoding peroxisome ascorbate peroxidase (APX) from *Salicornia brachiata* Roxb. was overexpressed in tobacco plants, the plants showed increased salt and drought tolerance compared to the wild type (Singh et al., 2014). Surender et al. (2015) found that the survival capability of transgenic *Sorghum bicolor* lines under salt stress was more enhanced when transformed with the mutated pyrroline-5-carboxylate synthetase gene, which encodes a key enzyme for glutamate proline synthesis and protects photosynthetic and antioxidant enzyme activities. Transformation of sugarcane plants with the *Vigna aconitifolia P5CS* gene not only conferred salt tolerance in transgenic lines but also the higher gene expression was accompanied by higher proline content, reduction of malondialdehyde, low accumulation of Na^+^, and sustained photochemical efficiency of photosystem II compared with the control (Guerzoni et al., 2014). This suggests that proline protects the photosynthetic system and prevents oxidative damage under salt stress. Ahmad et al. showed that the expression of the SOD, CAT, and APX genes in chickpea (*Cicer arietinum* L.) plants was upregulated, and the exogenous nitric oxide was also significantly increased under salt stress (Ahmad et al., 2016). Studying transgenic wheat plants containing the *mtlD* gene, El-yazal et al. found that transgenic plants had improved salt tolerance over non-transgenics, showing better growth traits, physio-biochemical attributes, and activities of antioxidant enzymes (El-Yazal et al., 2016).

In the present study, salt-tolerant *Tritipyrum* hybrid and salt-sensitive common wheat (“CS”) were evaluated for growth parameters, various biochemical attributes, and transcriptomic responses against salt stress and recovery. We found that salt stress inhibited the growth of the two cultivars as evidenced by examination of growth parameters. The RL and RDW of salt-tolerant “Y1805” showed no significant difference between the treatments and controls at each stage. Whereas those of salt-sensitive “CS” were significantly reduced by 10.84 and 22.35% of the controls at the R2 stage after recovery, salt stress had more inhibitory effects on the roots than the rest of the plant. “Y1805” represented stronger salt tolerance than “CS” according to its RL and plant dry weight. These results were in accordance with those of Basal et al. who observed a reduction in SDW and RDW with increasing salt levels in salt-tolerant and salt-sensitive cotton varieties (Basal et al., 2006). Moreover, the findings of Guo et al. corroborate our results and state that under salt stress, the dry matter accumulation rate of cotton varieties decreases, but it can be resumed in the salt-tolerant varieties after recovery (Guo et al., 2015).

Photosynthesis is the basis for the efficient conversion of light energy into the growth and development of plants. Leaf photosynthetic rate is an important indicator of photosynthesis, and the chlorophyll content of leaves is closely related to photosynthetic rate. Salt stress can lead to the decrease of chlorophyll content in plants, but the degree of Chl-a/b reduction will differ among different varieties (Ehsanzadeh et al., 2009; Sarwar and Shahbaz, 2019; Rehman et al., 2021). In this work, both the Pn and Chl-a/b levels of “Y1805” and “CS” were significantly affected by salt stress, though, the level of damage in the former was significantly less than in the latter, and these changes were subsequently reversed in “Y1805” during the recovery process. It was also shown that Chl-a was more sensitive to salt stress than Chl-b. At the T2 stage, the Chl-a content of “CS” decreased significantly by 26.07% compared with that of “Y1805.” These results were in agreement with previous reports (Muhammad et al., 2012; Abdellaoui et al., 2017), where salt stress and subsequent Na^+^ accumulation resulted in the degradation of photosynthetic pigment and Pn reduction in cactus and *Stipa lagascae* plants, respectively.

Salt stress induces the overproduction of ROS, which causes redox imbalance and oxidative damage in cells (Demiral and Türkan, 2005; Hossain and Dietz, 2016; Zhang et al., 2016; Ahmad et al., 2019). In response, antioxidant defense machinery is activated by various enzymatic and non-enzymatic antioxidants to alleviate this stress and scavenge ROS (Kakar et al., 2016, 2017; Vighi et al., 2017; Fan et al., 2020). SOD reacts with superoxide anion radical (O^2−^) to produce H_2_O_2_, while CAT is an H_2_O_2_ scavenging enzyme, which can decompose H_2_O_2_ and avoid the accumulation of H_2_O_2_ in cells. The combination of SOD and CAT can effectively reduce the harm of ROS in plant cells. In this study, the SOD and CAT activities of the two wheat varieties were significantly upregulated under salt stress, which was consistent with the results of Esfandiari et al. (2007). Under salinity, salt-tolerant varieties are more likely to activate SOD and CAT than other varieties (Al Kharusi et al., 2019). Here, “Y1805” enhanced the SOD and CAT activities rapidly under salt stress, and they were reduced quickly after recovery, indicating that its ROS scavenging ability was higher than in “CS.”

Cells accumulate free proline, soluble sugars, and other osmotic regulators under salt stress, which can regulate the osmotic potential of cells and maintain water balance. Proline accumulation is considered one of the important regulatory mechanisms for plants to adapt to saline and alkaline environment (Meena et al., 2019; Sharma et al., 2019), and its exogenous application has reportedly improved the salt tolerance of several crops (Sun and Hong, 2010; Nounjan et al., 2012; Sobahan et al., 2012; El Moukhtari et al., 2020). Moreover, proline is also a metal chelator, an antioxidative defense molecule, and a signaling molecule (Hayat et al., 2012). In this experiment, the ability of proline accumulation was different among the two varieties at different stages, where the proline in “Y1805” was accumulated at an increasing rate during each stage of salt stress, compared with “CS” that showed a zigzag trendline. Previously, Sivakumar et al., showed that the proline content of a few tomato varieties did not increase significantly after 24 h of short-term stress or under low salinity (Sivakumar et al., 2019). The proline content of “Y1805” began to rise at the T2 stage of salt stress and peaked at the R1 stage, and was 2.00-fold higher than that of “CS.” These observations suggest that “Y1805” could synthesize more proline than “CS” to cope with salt stress damage. Soluble sugars, as osmotic regulators and signal molecules, participate in the response and adaptation of plants to environmental stress (Wang et al., 2018) and can be used to identify salt tolerance in plants (Bai et al., 2013). In a study conducted by Kerepesi and Galiba, the soluble sugar content of the salt-tolerant wheat variety “Sakha” was higher than that of “CS” under salt stress (Kerepesi and Galiba, 2000). Similar results were obtained in this study. The soluble sugar content in “Y1805” increased significantly from the T2 stage, and kept stable until the R1 stage, and returned to the control level at the R2 stage after recovery. In contrast, “CS” showed little change in soluble sugar content during salt stress and only showed an increase after recovery, indicating that “Y1805” had strong soluble sugar regulatory abilities.

In this study, we used a sequential GISH-FISH technique to determine the genomic composition of salt-tolerant *Tritipyrum* “Y1805” and identified 56 chromosomes. It was revealed that the “Y1805” genome comprised A, B, and D chromosomes from wheat parents, but also contained the E genome of chromosomes that originated from *T. elongatum*, adding to its genetic diversity and suitability as a hybrid with salt-tolerant traits.

Comparison of the DEGs between the two cultivars directly reflects the mechanism of plant salt tolerance. In this study, a total of 112,454 unigenes were obtained by RNA-seq analysis, including 44,886 DEGs. Transcriptome analysis showed that the differences in the transcriptional regulatory networks of salt-tolerant and salt-sensitive germplasm are not only in response to salt stress but also in recovery. The number of DEGs in each variety and treatment varied. Among these DEGs, some were expressed solely against salt stress or recovery, while others were co-expressed mutually between “Y1805” and “CS.” The DEGs of “Y1805” at the T2 stage were enriched in several photosynthetic GO terms and KEGG pathways related to “fatty acid biosynthesis and metabolism.” The results of Luo et al. showed that salt-tolerant wheat could enhance the photosynthetic system and improve the salt tolerance of wheat through pathways related to polyunsaturated fatty acid metabolism (Luo et al., 2019). In this experiment, it was found that there were more DEGs related to “fatty acid” and “photosynthesis” in “Y1805” under salt stress, but expression decreased rapidly after recovery. It may be that “Y1805” increased salt tolerance through fatty acid synthesis and metabolism and ensured that the photosynthetic machinery of the cells was actively regulated under salt stress, and remained almost intact during the recovery process. “Y1805” was enriched in the metabolic pathway of “start and conquer metabolism” at the T2 stage, and in “glutathione metabolism” and “arginine and proline metabolism” at the T2 and R1 stages, respectively. Starch and sucrose metabolism is considered to determine the soluble sugar content in plants and affect osmotic regulation (Balibrea et al., 2006). Differences in expression levels of related DEGs under salt stress will lead to differences in soluble sugar contents in the plant materials studied (Wei et al., 2020). The transcriptional level of enzymes related to glutathione biosynthesis and glutathione content can alleviate the effects of salt stress on photosynthesis (Nazar et al., 2015), maintain the dynamic balance of cell redox (Zhou et al., 2017), and reduce the toxicity of methylglyoxal induced by salt stress (Khunpon et al., 2018). Amirbakhtiar et al. (2019) also reported that DEGs in salt-tolerant bread wheat were enriched in “transporters,” “phenylpropanoid biosynthesis,” “transcription factors,” “glycosyltransferases,” “glutathione metabolism,” and “plant hormone signal transduction” pathways during salt stress, which also support our results that these are significant pathways in the abiotic and biological stress response in plants.

“Arginine and proline metabolism” is the key pathway for proline accumulation in plants under salt stress, and as mentioned earlier, proline is an important osmotic regulator for salt tolerance (Sakr et al., 2012). Here, the high abundance of DEGs at the T2 and R1 stages indicated that the expression of DEGs in “Y1805” may be one of the important reasons for its high salt tolerance. The KEGG pathway enrichment analysis of “Y1805” revealed that DEGs at the T2 stage were significantly enriched in “carbohydrates,” “lipids,” and “amino acids metabolism” along with “signal transduction,” “environmental adaptation,” “secondary metabolites biosynthesis,” and “membrane transport.” At the R1 stage, DEGs were enriched in “glycan biosynthesis and metabolism” and “carbohydrate metabolism.” There were significant differences in the metabolic pathways regulated by DEGs in “Y1805” under salt stress and recovery conditions, which indicated that there were differences in the regulatory mechanisms of “Y1805” in response to salt stress and recovery.

Lastly, the expression patterns of salt stress and recovery responsive DEGs in “Y1805” were determined. The results indicated that the functions of many salt stress and recovery responsive DEGs in “Y1805” were closely correlated with the levels of SOD and CAT activities, as well as the levels of sugar, proline, chlorophyll, and Pn. These DEGs played key roles in the KEGG pathways of “peroxisome” (ko04146), “arginine and proline metabolism” (ko00330), “starch and sucrose metabolism” (ko00500), “chlorophyll and porphyrin metabolism” (ko00860), and “photosynthesis” (ko00195), respectively. Functional analysis revealed that these genes are involved in specific metabolic pathways and mechanisms, which might have a unique significance in wheat salt tolerance. After recovery, some genes were potentially upregulated at the R1 stage, thus contributing to the recovery of wheat growth and development. Through transcriptome analysis, it was found that “Y1805” had many specific DEGs under salt stress and recovery. These DEGs were enriched in multiple related pathways, and their enrichment differed under salt stress and recovery conditions, showing that plants can regulate specific DEGs under different conditions. Finally, the patterns of the up/downregulated genes combined with the detection of related growth indicators prompted us to conclude that the function of many stress-responsive DEGs is closely related to the activities of antioxidant enzymes and the levels of sugar, proline, chlorophyll, and Pn.

## Conclusion

Salt-tolerant *Tritipyrum* (“Y1805”) and salt-sensitive wheat (“CS”) were chosen from 121 wheat germplasms using salt-tolerant experiments. Overall, 56 chromosomes were identified in “Y1805” using sequential GISH-FISH analysis, which comprised A, B, and D chromosomes from common wheat parent and E genome chromosomes that originated from *T. elongatum*, which added to its genetic diversity and salt-tolerant traits. Growth parameters revealed that salt stress had a greater inhibitory effect on the roots than on the shoots, and “Y1805” demonstrated stronger salt tolerance than “CS.” “Y1805” could enhance the antioxidant activity more rapidly than “CS” under salt stress to protect the cells from damage caused by ROS. “Y1805” could synthesize more proline and soluble sugars than “CS’ to cope with salt stress damage. Both Pn and Chlorophyll a/b contents were affected by salt stress, although the level of damage in “Y1805” was significantly lesser than in “CS.” Using transcriptome analysis, the functions of many salt-responsive DEGs were closely correlated with “peroxisome,” “arginine and proline metabolism,” “starch and sucrose metabolism,” “chlorophyll and porphyrin metabolism,” “photosynthesis,” and “fatty acid biosynthesis and metabolism” KEGG pathways. The strong salt tolerance of “Y1805” may be mainly attributed to ROS scavenging, osmoregulation, ion homeostasis, cell wall remodeling, and signal transduction. Some novel candidate genes related to antioxidase, osmoregulator, or photosynthesis pathways were also found under salt stress in “Y1805.” The outcomes of growth and physio-biochemical analyses were consistent with the transcriptome data. The nature of the salt tolerance mechanisms –*per se*- is already known in other plant species and but seems to be more quantitatively effective in the tolerant wheat genotype. These findings provide useful information for the cultivation and breeding of salt-tolerant wheat.

## Data Availability Statement

The datasets presented in this study can be found in online repositories. The raw sequence reads were deposited into NCBI SRA database under accession no. PRJNA769794 (https://www.ncbi.nlm.nih.gov/sra/?term=PRJNA769794).

## Author Contributions

ZL, RX, and SZ designed the experiments. Material preparation, data collection, and analysis were performed by ZP, YW, RY, and ZY. ZP, YW, GG, QZ, KK, ZL, and SZ wrote the manuscript. All authors have read and approved the final manuscript.

## Funding

This study was financially supported by the National Natural Science Foundation of China (31860380 and 32160442) and the Science Foundation of Guizhou Province [(2018)5781 and (2019)1110].

## Conflict of Interest

The authors declare that the research was conducted in the absence of any commercial or financial relationships that could be construed as a potential conflict of interest.

## Publisher’s Note

All claims expressed in this article are solely those of the authors and do not necessarily represent those of their affiliated organizations, or those of the publisher, the editors and the reviewers. Any product that may be evaluated in this article, or claim that may be made by its manufacturer, is not guaranteed or endorsed by the publisher.
